# Ambient ozone pollution impairs glucose homeostasis and contributes to renal function decline: Population-based evidence

**DOI:** 10.1016/j.ecoenv.2023.115803

**Published:** 2024-01-01

**Authors:** Shouxin Peng, Bingbing Chen, Zhaoyuan Li, Jinhui Sun, Feifei Liu, Xiaoyi Yin, Yi Zhou, Huanfeng Shen, Hao Xiang

**Affiliations:** a Department of Global Health, School of Public Health, Wuhan University, Wuhan 430071, Hubei, PR China; b Global Health Institute, Wuhan University, Wuhan 430071, Hubei, PR China; c School of Resource and Environmental Sciences, Wuhan University, Wuhan 430079, Hubei, PR China

**Keywords:** Ozone pollution, Renal function, Mediation effect, Insulin resistance, Hyperglycemia

## Abstract

Particulate matter pollution could increase the risk of kidney disease, while evidence for ozone exposure is less well-established. Here, we aimed to evaluate the effect of ozone pollution on renal function and explore mechanisms. We first conducted a cross-sectional study based on Wuhan Chronic Disease Cohort Study baseline information. We recruited 2699 eligible participants, estimated their residential ozone concentrations, collected fasting peripheral blood samples for biochemical analysis and calculated the estimated glomerular filtration rate (eGFR). The linear regression model was applied to evaluate the long-term association between ozone pollution and eGFR. Then, we recruited another 70 volunteers as a panel with 8 rounds follow-up visits. We calculated the eGFR and measured fasting blood glucose and lipid levels. The linear mixed-effect model along with mediation analysis were performed to confirm the short-term association and explore potential mechanisms, respectively. For the long-term association, a 10.95 μg/m^3^ increment of 3-year ozone exposure was associated with 2.96 mL/min/1.73 m^2^ decrease in eGFR (95%CI: −4.85, −1.06). Furthermore, the drinkers exhibited a pronounced declination of eGFR (−7.46 mL/min/1.73 m^2^, 95%CI: −11.84, −3.08) compared to non-drinkers in relation to ozone exposure. Additionally, a 19.02 μg/m^3^ increase in 3-day ozone concentrations was related to 2.51 mL/min/1.73 m^2^ decrease in eGFR (95%CI: −3.78, −1.26). Hyperglycemia and insulin resistance mediated 12.2% and 16.5% of the aforementioned association, respectively. Our findings indicated that higher ozone pollution could affect renal function, and the hyperglycemia and insulin resistance linked to ozone might be the underlying mechanisms.

## Highlights

• Both long-term and short-term ozone exposure was associated with eGFR decline.
• Drinkers showed higher risk estimate.
• Hyperglycemia and insulin resistance might be potential mechanisms.
• Ambient ozone concentrations were higher than the personal exposure levels.

## Graphical Abstract

**Figure:**
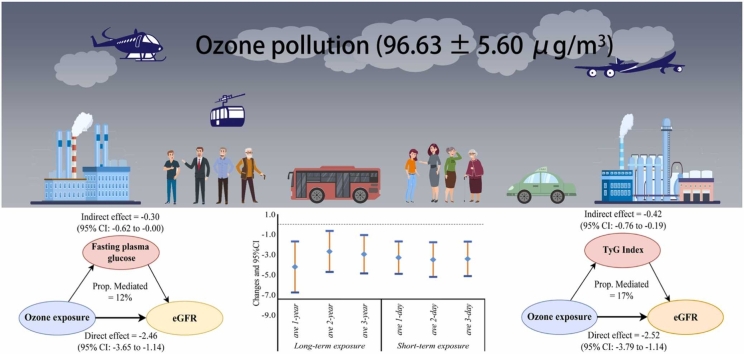

## 1 Introduction

Over the past decade, global particulate matter pollution has improved profoundly under the implemented effective air pollution control measures (Zhang et al., 2019). Simultaneously, global ozone concentration increased rapidly, driven by the environmental pollution sources transformation and climate change (Orru et al., 2017, Xiao et al., 2022). It was estimated that ozone pollution contributes to more than 1 million premature deaths and 4.116 million DALYs worldwide each year (GBD, 2016, Im et al., 2023). The health hazards of ozone exposure have attracted great concern. Previous evidence suggested that air pollution, especially particulate matter, adversely affected renal function and also contributed to kidney disease (Tavera Busso et al., 2018, Ye et al., 2021). However, it remains uncertain whether ozone pollution could also affect renal function.

As a gaseous pollutant, the health effects and pathogenic mechanisms of ozone might differ from those of particulate matter (Rivas-Arancibia et al., 2022, Shinyashiki et al., 2009). Currently, available evidence for ozone pollution and renal function differed significantly (Table S1). A cohort study including 5090 African American participants observed that 3-year ozone exposure was associated with decreased eGFR (0.30 mL/min/1.73 m^2^, 95%CI: −0.60, −0.04) (Weaver et al., 2019). In contrast, another cohort study in Thailand reported an insignificant association (Paoin et al., 2022). However, the CHCN-BTH cohort found a positive association between ozone pollution and renal function with increased eGFR of 1.151 mL/min/1.73 m^2^ (Wen et al., 2023). Reasons for the inconsistency were complex, and several plausible explanations included the difference in ozone concentrations, genetic causes, kidney-disease risk factors, lifestyles, or culture. Generally, current evidence on ozone pollution and renal function remained scarce and heterogeneous. Further studies were required.

Previous research has determined oxidative stress damage and systemic inflammation response as the two main pathways for cardiometabolic damage in response to ozone pollution (Arjomandi et al., 2018, Jantzen et al., 2018). Subsequently, the injured cardiometabolic function, such as hyperglycemia or blood pressure, could impair the renal vasculature and lead to a decrease in eGFR. Specifically, a panel study among 166 older adults reported that exposure to ozone was positively associated with fasting plasma glucose (FPG) (Li et al., 2021b). Another research among 1112 healthy adults also observed that hyperglycemia was related to the decreased eGFR (Sun et al., 2015). Homoplastically, findings from the Veterans Administration Normative Aging Study reported that exposure to fine particulate matter (PM_2.5_) was related to increased diastolic blood pressure, which further contributed to lower eGFR (Mehta et al., 2016). However, the bio-mechanisms for ozone exposure provoke renal function declination by affecting FPG or blood pressure was not well-established.

Hence, we conducted a cross-sectional study and a longitudinal panel study to explore the effect of ozone pollution on renal function. Besides, we leveraged the strength of longitudinal study in causal inference, and incorporated the causal mediated effect model to explore potential mediating pathways. Our results will contribute to the evidence between ozone exposure and renal function by synthesizing multiple studies.

## 2 Methods

### 2.1 Study design and participants

We evaluated the chronic effect of ozone exposure on renal function based on baseline information from the Wuhan Chronic Disease Cohort Study (WCDCS) established in 2019. Detailed information for WCDCS has been described elsewhere (Chen et al., 2022, Wang et al., 2022b). Briefly, we employed a multi-stage probability proportionate to size method to recruit a total of 10,253 participants. Among these, a subgroup of 2747 participants (25–30% of the whole cohort) from 20 communities was further selected using the stratified cluster random sampling method according to the counties and the proportion of participants (Fig. S1). All the selected participants underwent physical examination, peripheral venous blood collection and serum biochemical analysis. Additionally, personal demographic characters, behavioral factors, and health status were collected through structured questionnaires. Currently, our research was based on the above subgroup with 2699 participants, after excluding the subjects who lacked key information on biochemical data or behavioral lifestyle (n = 48).

Acute effect of ozone exposure on renal function was focused on another longitudinal panel study (Li et al., 2023, Peng et al., 2022a). Briefly, a group of 70 healthy adults were scheduled for 8 rounds of follow-up visits with peripheral venous blood and behavioral lifestyles collection from September 2019 to January 2020. A detailed description was offered in the Supplement eMethods. All participants were required to record their indoor and outdoor activity time during the past 3 days prior to each follow-up visit. Totally, 533 visit records were finally included in the current analysis. All the above participants were provided written informed consent, and the research design was approved by the Medical Ethical Committee of Wuhan University.

### 2.2 Environment exposure

Long-term ozone exposure concentrations for the WCDCS were evaluated using the ChinaHighAirPollutants (CHAP) with temporal and spatial resolutions of 1 day and 10 km, respectively (Wei et al., 2022). Briefly, an extended spatial-temporal extreme random tree model was developed to estimate surface ozone by integrating groups of key environmental parameters. A 10-fold cross-validation showed the estimated model has good predictive ability with R^2^ and root-mean-square error for daily predictions of 87% and 17.10 μg/m^3^ (Wei et al., 2022). Participants' resident addresses, detailed to the community, were geocoded into latitude and longitude data using Baidu Map API, and then mapped to the CHAP dataset. Short-term ozone exposure concentrations for the later panel study were obtained from the nearest Donghu Liyuan air monitoring station, which was located within 2 km of the participants' activity areas (Peng et al., 2022b). Outdoor ozone exposure was directly approximated by the monitoring station observations. The indoor exposure was estimated using the multiplicative term of outdoor ozone concentration and the infiltration factor for Hubei constructed previously (0.27 for summer, 0.18 for winter, 0.23 for spring and autumn) (Hu et al., 2020). We then calculated the adjusted personal ozone exposure concentrations by adding the outdoor and indoor exposure, according to the self-reported time-activity diary (Wang et al., 2022a). Currently, 1–3-year average ozone concentrations before the recruitment were defined as long-term exposure, while 1–3-day average before each physical examination were considered as short-term exposure. Besides, the single-day lag patterns of effects related to short-term ozone exposure were also performed.

### 2.3 Outcome assessment

At each interview, 10 mL of fasting peripheral venous blood was drawn from all participants by a medical professional. A total of 3280 blood samples, 2747 from the WCDCS and 533 from the panel study, were centrifuged into serum and plasma and ultimately stored at − 80 ºC. We performed a full-automatic biochemical analyzer (Hitachi 7600, Hitachi Co., Tokyo, Japan) to determine serum creatinine (sCr) concentration. We then calculated the eGFR based on the modified Modification of Diet in Renal Disease (MDRD), which was more appropriate for Chinese residents (Ma et al., 2006). The plasma glucose and serum triglyceride concentrations were determined by enzymatic hexokinase method using an automatic biochemical analyzer (Cobas c701, Roche, Japan). Then, we calculated the triglyceride-glucose (TyG) index, which was determined as a surrogate indicator for insulin resistance (Simental-Mendía et al., 2008). Participants' blood pressure values were measured by electronic blood pressure monitors (Omron U10L, OMRON Healthcare Co., Ltd., China).

### 2.4 Covariates

Groups of covariates were prior-selected based on published research (Rasking et al., 2022, Ye et al., 2021). Demographic characters included age (continuous, years), gender (male or female), BMI (continuous, kg/m^2^), marriage status (married or cohabitation, unmarried/separated/divorced/widowed), education status (middle school or lower, junior college or higher), and employment (official or worker, retiree or others). Lifestyle factors included smoking status (non-smoker, smoker: smoking more than once a week for more than 6 months), drinking status (non-drinker, drinker: drinking alcohol more than once a week) and physical exercise (no, yes: 30 min of regular physical activities more times 3 times a week). The criteria for defining hypertension include: (i) systolic blood pressure > 140 mmHg, (ii) diastolic blood pressure > 90 mmHg, (iii) diagnoses of hypertension by a clinical doctor, and/or (iv) taking anti-hypertensive medication. We defined diabetes as the FPG greater than 200 mg/dL, clinically diagnosed with diabetes and/or intake of antidiabetic medication. Additional covariates from the panel study included sleep status, categorized as either "no" or "yes" (indicating average daily sleep over 7 h in the past week).

### 2.5 Statistical analysis

Characters involving demographic factors and the health status of the overall participants were described as frequency and percentage. We performed a multi-variable linear regression model to explore the long-term association between ozone exposure and eGFR. We primordially developed model 1, which merely included ozone. In the transitional model 2, we further adjusted for age, gender, BMI. On the basics of model 2, we adjusted hypertension and diabetes. We fully adjusted the smoking, drinking status, education, marriage and employment along with the aforementioned covariates in the model 4. The variance inflation factors of all variables were no more than 2 in the fully adjusted model, suggesting there was no potential multi-collinearity problem. Additionally, we performed the subgroup analysis by age (<45, 45–65 or ≥ 65 years), gender, BMI (< 24 or ≥ 24 kg/m^2^, signifying overweight or higher) (Zhou, 2002), lifestyles (drinking, smoking) and health status (hypertension, diabetes), which were related to kidney disease in previous research (Li et al., 2021d, Yang et al., 2022). The Z-test was employed to examine statistical differences between groups (Altman and Bland, 2003).

We also conducted the linear mixed-effect model (LME) with the participant ID as the fixed effect to examine the acute adverse effect for personal ozone pollution on renal function (Supplement eMethods). We firstly built a crude model without adjusting for any confounding factors, and further adjusted age, gender, BMI, exercise, sleep status, smoking and drinking into the main model (Model 5). The exposure-response curves between 3-day average personal ozone concentrations and eGFR were generated using restricted cubic splines with three degrees of freedom. Besides, we performed a causal mediation analysis (Qin and Wang, 2023), following the Baron-Kenny’s step, to examine whether elevated FPG, TyG index or blood pressure could be mechanisms through which ozone exposure affected renal function (Supplement eMethods). In brief, we initially developed a LME model to determine the association between ozone exposure and potential bio-mediators (FPG, blood pressure, TyG index). The significant bio-mediators were further adjusted for the association between ozone exposure and eGFR. We then performed the mediation analysis using the R packages of “mediation” with bootstrapping of 1000 simulations.

We also performed several sensitivity analyses to examine the robustness of our findings. Firstly, we further adjusted for PM_2.5_ and normalized difference vegetation index (NDVI) as covariates in the long-term association of ozone exposure with eGFR to exclude the effect of residential greenness. Secondly, we further adjusted for household fuel type (solid fuels or clean fuels) in the long-term association analysis, as it has been reported that fuel type could affect renal function (Xue et al., 2022). Thirdly, we defined the renal function decline based on eGFR less than 60 mL/min/1.73 m^2^. The logistic regression models were conducted to examine the long-term association. Finally, we further adjusted the PM_2.5_, ambient temperature or relative humidity in the short-term association between ozone and eGFR, respectively. The estimates of the association were presented with changes in eGFR with an interquartile range (IQR) increase in ozone concentrations. All the above statistical analysis operations were completed in R 4.2.0, and the statistical significance was defined as a two-sided p-value less than 0.05.

## 3 Results

### 3.1 Characteristics of participants and ozone pollution

A total of 2769 participants, involving 2699 from the WCDCS and 70 from the later panel study, were included into our current analyses (Fig. S2). The average age of 53.01 (13.87) and 20.40 (1.53) years, respectively. The BMI of the participants in the two studies was 24.53 (3.55) and 21.50 (2.75) kg/m^2^, respectively. A majority of the study participants in the WCDCS were female (64.1%), married (86.0%), non-smokers (80.2%), non-drinkers (77.5%), and had junior high school or less education (78.7%). The prevalence of hypertension and diabetes was 37.3% and 13.3%, respectively (Table 1). The average ambient ozone concentration was 96.63 (5.60) μg/m^3^. Besides, there were 14 males and 56 females in the later panel study, and 533 blood samples were collected. The average daily sleep duration of the participants was 7.82 (1.16) hours, while the average outdoor time was 3.08 (1.81) hours over the 533 observations (Table S2). The outdoor ozone concentrations were 48.20 (40.00) μg/m^3^, and the individual exposure concentration, weighted by activity patterns, was 16.00 (14.70) μg/m^3^ over the duration of all eight follow-up visits.

**Table 1:** Baseline characteristics (n%) for the study population in WCDCS.

Variable		All participants (n = 2699)
Gender		
	Male	970 (35.9%)
	Female	1729 (64.1%)
Age, years		
	< 45	676 (25.1%)
	45–65	1429 (52.9%)
	≥ 65	594 (22.0%)
BMI, kg/m^2^		
	< 24	1225 (45.4%)
	≥ 24	1474 (54.6%)
Marital status		
	Unmarried and other	377 (14.0%)
	Married	2322 (86.0%)
Education		
	Middle or below	2123 (78.7%)
	College or above	576 (21.3%)
Smoking		
	Smoker	534 (19.8%)
	Non-smoker	2165 (80.2%)
Drinking		
	Drinker	607 (22.5%)
	Non-drinker	2092 (77.5%)
Hypertension		
	Yes	1006 (37.3%)
	No	1693 (62.7%)
Diabetes		
	Yes	358 (13.3%)
	No	2341 (86.7%)
Employment		
	Official or worker	764 (28.3%)
	Retiree and other	1935 (71.7%)

Abbreviations: WCDCS, Wuhan Chronic Disease Cohort Study; BMI, body mass index.

### 3.2 Estimated long-term association between ozone and renal function

**Fig. 1:**
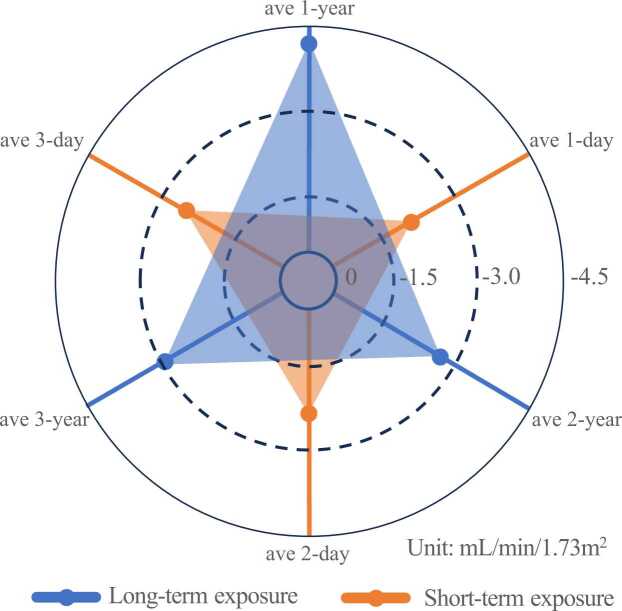
Radar chart of ozone effects on eGFR for long-term (10.95 μg/m^3^) and short-term (19.02 μg/m^3^ increment) exposure. Abbreviation: eGFR, estimated glomerular filtration rate.

**Fig. 2:**
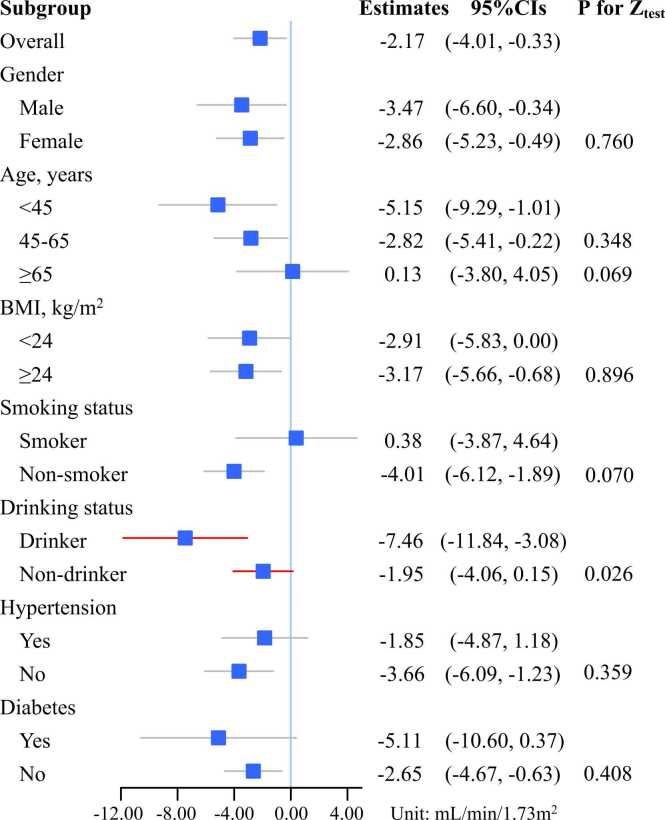
The stratified analyses of the association between long-term ozone exposure and eGFR. Abbreviations: eGFR, estimated glomerular filtration rate; CI, confidence interval; BMI, body mass index.

We observed negative association between long-term ozone exposure and eGFR (Fig. 1). Specifically, an IQR (10.95 μg/m^3^) increment in 3-year ambient ozone concentrations was associated with 4.21 mL/min/1.73 m^2^ (95%CI: −6.74, −1.69) decrease in eGFR after fully adjusting other covariates (Table 2). Similar negative associations were also found at the 1- and 2-year exposure windows, − 2.96 (95%CI: −4.85, −1.06) and − 2.68 (95%CI: −4.71, −0.65) mL/min/1.73 m^2^, respectively. Subgroup analysis showed greater associations in drinkers (β = −7.46, 95%CI: −11.84, −3.08) than non-drinkers (β = −1.95, 95%CI: −4.06, 0.15). We also found greater associations in non-smokers (β = −4.01, 95%CI: −6.12, −1.89) than smokers (β = 0.38, 95%CI: −3.87, 4.64), but the difference was insignificant (*p* = 0.07) (Fig. 2).

**Table 2:** Association between long-term ozone exposure and eGFR. Estimates and 95%CI per IQR (10.95 μg/m^3^) increment. (mL/min/1.73 m^2^).

	Ave 1-year	Ave 2-year	Ave 3-year
Model 1^a^	-7.70 (−10.28, −4.93)	-5.53 (−7.65, −3.42)	-5.77 (−7.74, −3.80)
Model 2^b^	-3.32 (−5.81, −0.83)	-1.91 (−3.88, 0.07)	-2.28 (−4.12, −0.43)
Model 3^c^	-3.29 (−5.77, −0.80)	-1.82 (−3.79, 0.15)	-2.17 (−4.01, −0.33)
Model 4^d^	-4.21 (−6.74, −1.69)	-2.68 (−4.71, −0.65)	-2.96 (−4.85, −1.06)
+ fuel type^e^	-5.02 (−7.84, −2.19)	-3.41 (−5.72, −1.10)	-3.74 (−5.89, −1.59)
+ PM_2.5_	-3.85 (−6.46, −1.25)	-2.66 (−4.87, −0.46)	-3.46 (−5.54, −1.39)
+ NDVI	-6.09 (−9.14, −3.04)	-4.13 (−6.81, −1.46)	-4.63 (−7.16, −2.10)

Abbreviation: eGFR, estimated glomerular filtration rate; IQR, interquartile range; CI, confidence interval; PM_2.5_, fine particulate matter (μg/m^3^); NDVI, normalized difference vegetation index.

a Model 1 was the crude model.

b Model 2 adjusted for age, gender, BMI.

c Model 3 plus adjusted for hypertension and diabetes.

d Model 4 was the fully adjusted model, which further adjusted for education status, occupation, smoking status, drinking status and marriage status.

e Fuel type include solid fuels or clean fuels with 318 missing data.

### 3.3 Estimated short-term association between ozone and renal function

We observed the negative and non-linear association between short-term ozone exposure and eGFR, and slope was slower over higher concentrations (Fig. S3). An IQR (19.02 μg/m^3^) increment in averaged 3-day MDA8 ozone concentrations was associated with 2.51 mL/min/1.73 m^2^ (95%CI: −3.78, −1.26) decrease in eGFR. Similar negative associations were also found at the 1- and 2-day exposure windows, − 2.09 (95%CI: −3.28, −0.90) and − 2.35 (95%CI: −3.62, −1.10) mL/min/1.73 m^2^, respectively (Table 3). Our results also suggested a greater effect of decreasing eGFR with increasing ozone exposure time. For the lag effect of ozone pollution, we observed the lag 2 day exposure showed larger risk estimate with decreased eGFR of − 2.76 mL/min/1.73 m^2^ (95%CI: −3.92, −1.60) (Table S3).

**Table 3:** Association between short-term ozone exposure and eGFR. Estimates and 95%CI per IQR (19.02 μg/m^3^) increment. (mL/min/1.73 m^2^).

	Ave 1 day	Ave 2 days	Ave 3 days
Model 1^a^	-2.36 (−3.52, −1.20)	-2.64 (−3.87, −1.41)	-2.79 (−4.02, −1.56)
Model 5^b^	-2.09 (−3.28, −0.90)	-2.35 (−3.62, −1.10)	-2.51 (−3.78, −1.26)
+PM_2.5_	-2.19 (−3.46, −0.93)	-2.01 (−3.38, −0.63)	-2.42 (−3.87, −0.96)
+AT	-3.29 (−4.90, −1.69)	-3.49 (−5.20, −1.78)	-3.41 (−5.11, −1.71)
+RH	-2.28 (−3.48, −1.09)	-2.56 (−3.83, −1.29)	-2.73 (−4.00, −1.45)

Abbreviation: eGFR, estimated glomerular filtration rate; IQR, interquartile range; CI, confidence interval; PM_2.5_, fine particulate matter (μg/m^3^); AT, ambient temperature (°C); RH, relative humidity (%).

a Model 1 was the crude model.

b Model 5 adjusted for age, gender, BMI, physical exercise, smoking, drinking and sleep duration.

### 3.4 Mechanisms for ozone pollution on renal function

**Fig. 3:**
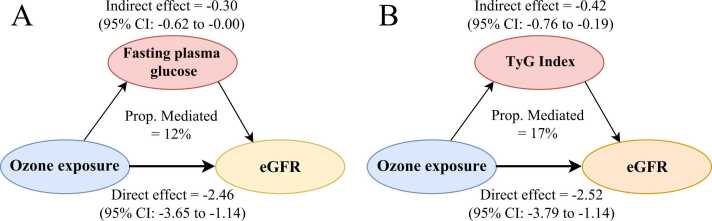
Mediation analysis of fasting plasma glucose and TyG index on eGFR after ozone exposure. Abbreviations: TyG index, triglyceride-glucose index; eGFR estimated glomerular filtration rate.

We initially examined the association between ozone exposure and mediators under a 3-day average exposure window, which showed the most detrimental effects on renal function. We observed an IQR increment in ozone was related to 0.11 mmol/L (95%CI: 0.07, 0.15) and 0.06 (95%CI: 0.04, 0.09) increase in FPG and TyG index, respectively. However, the effect of ozone exposure on blood pressure indicators were insignificant. Hence, we further examined the mediation effect of FPG and TyG index as potential bio-mediators. It was estimated that the FPG and TyG index contributed 12.2% and 16.5% associations between ozone exposure and eGFR decline, respectively (Fig. 3). The natural indirect effects for FPG and TyG index were − 0.30 (95%CI: −0.60, 0.00) and − 0.42 (95%CI: −0.76, −0.19) mL/min/1.73 m^2^, respectively.

### 3.5 Sensitivity analysis

We also conducted series of sensitivity analysis to examine our results robustness. We firstly adjusted for PM_2.5_, NDVI_500 m_ and household fuel type as additional covariates, respectively. The negative association between ozone exposure and eGFR remain significant (Table 2). We also observed a positive association between renal function decline (eGFR < 60 mL/min/1.73 m^2^) and ozone exposure with the adjusted OR of 1.06 (95%CI: 1.02, 1.10) (Table S4). Finally, we further adjusted for PM_2.5_, ambient temperature or relative humidity, and the negative association between short-term ozone exposure and eGFR remained significant (Table 3). All the above analysis demonstrated the robustness of our results.

## 4 Discussion

Our current study is, to the best of our knowledge, the first population-based evidence to assess the effects of ozone exposure on renal function with potential mechanisms. We found both long-term and short-term ozone pollution were related to renal function declination. Furthermore, the drinkers were more prone to suffer greater risk estimates under higher ozone exposure scenario. We also observed the elevated FPG and insulin resistance could be bio-mechanisms for the above association. Our findings appeal to the public emphasize ambient ozone pollution. Another view also suggests that the interventions for insulin resistance might improve the renal function declination from ozone exposure.

Ambient ozone pollution levels have continued to rise over the past decade in China (Xiao et al., 2022). Wuhan was also a Chinese city with severe ozone pollution, as shown by the national distribution map of ozone exposure concentration (Liu et al., 2021, Xiao et al., 2022). The 1-year (Aug. 2018 to Jul. 2019) averaged ozone concentration was 107.20 μg/m^3^ for the current WCDCS participants, which significantly higher than the national average for the same period. In the later panel study, we observed the ambient ozone concentrations was about three times higher than the personal levels, which was similar with a previous panel study in Shanghai (Niu et al., 2018). Therefore, more accurate exposure assessment methods for ozone need to be developed and applied in environmental health research.

Previous research has successively indicated that long-term exposure to air pollutants could affect renal function or kidney diseases (Duan et al., 2022, Li et al., 2021d, Rasking et al., 2022, Ye et al., 2021). However, previous studies mainly focused on particulate matter, which was considered as the primary air pollutant for a prolonged period (Fang et al., 2020, Li et al., 2021c, Li et al., 2021d, Liu et al., 2020, Mehta et al., 2016). The detrimental effect of ozone was also more easily neglected as a kind of pollutant under the “blue sky” scenario. As demonstrated in large multicenter population-based evidence from China, long-term exposure to PM_1_ or PM_2.5_ was associated with a 0.37–0.99% decrease in eGFR (Li et al., 2021d). Our findings further revealed that ozone exposure could also cause a decrease in renal function drawing on particles research lines. Of the mere studies available, three had findings consistent with us. Thereinto, the Jackson Heart Study, which recruited 5090 adults with averaged age of 55.4 years, reported the inverse association between 1- or 3-year ozone exposure and eGFR (Weaver et al., 2019). Another national cross-sectional study also reported that a 10 μg/m^3^ increment in ozone concentrations was associated with the increased risk of CKD prevalence (OR = 1.11, 95%CI: 1.03, 1.21) (Yang et al., 2022). Additionally, a nationwide time-series study found the ozone showed pronounced impacts on emergency room visits for total kidney disease and acute kidney injury with attributable fraction of 2.3% and 5.9%, respectively (Lee et al., 2022). However, in addition to the three aforementioned studies, other studies on the association of ozone exposure with renal function or kidney disease were all insignificant (Hwang et al., 2021, Li et al., 2021a, Li et al., 2022, Paoin et al., 2022, Wang et al., 2020). Besides, Liu et al. reported that ambient ozone exposure might decrease the risk of developing CKD in a 2-year cohort study (Liu et al., 2022). We speculate that the inconsistent evidence may be due to the differences in study participants or ozone exposure concentrations. Generally, evidence for the association between ozone exposure and renal function was scarce, and more studies are needed to confirm the nephrotoxicity of ozone exposure.

We observed the drinker and the non-smoker were more vulnerable to the adverse effect of ozone exposure on renal function, although the stratified difference for smoking was insignificant (*P*_smoking_ = 0.07). Alcohol consumption was recognized as a traditional risk factor for kidney damage (Yamamoto et al., 2023). Evidence from animal experiments revealed that alcohol could induce inflammation injury, oxidative stress damage or disturb the immunoreaction, which further impair renal function (Harris et al., 2015, Latchoumycandane et al., 2015). About 10% of alcohol was excreted directly through kidneys, which could also cause tubular cell degeneration and glomerular dilation. We speculate that the aforementioned pathways may enhance the detrimental effects of ozone exposure. Smoking, also a well-documented risk factor for kidney disease (Kelly et al., 2021), might shelter the effect of ozone exposure as it shares similar exposure pathways from the respiratory trac. However, the evidence from our cross-sectional study should be also interpreted cautiously.

Currently, the mechanism evidence for the association between ozone exposure and renal function was almost blank. From the viewpoint of cardio-metabolism disorder, we observed that higher ozone exposure could elevate the FPG and increase insulin resistance which further risks renal function. Similarly, evidence from a birth cohort and a panel study both reported that exposure to ozone was positively associated with increased FPG and insulin resistance (Li et al., 2021b, Zhang et al., 2023). As previously revealed in animal experiments, ozone exposure in rats induces oxidative stress, endoplasmic reticulum stress and skeletal muscle insulin signal disruption, which in turn contribute to insulin resistance (Vella et al., 2015). Given that the kidney was a highly vascularized organ, impaired glucose homeostasis can easily cause an imbalance for the renal microenvironment. Increased insulin resistance could induce renal vasodilatation, which leads to impaired glomerular endothelial function and thus causes kidney injury (Groop et al., 2005, Ren et al., 2023). Besides, insulin resistance can provoke inflammation and induce overproduction of reactive oxidative stress, which in turn results tubulointerstitial damage and renal tissue fibrosis, respectively (Ren et al., 2023). Nevertheless, the above evidence appears to plausibly explain our population-based epidemiological findings. More experimental research should be conducted to elucidate the mechanism for ozone exposure and renal function impairment.

Our current research has several strengths. Firstly, we supplied the epidemiological evidence for the association between ozone exposure and renal function in both chronic and acute terms from cross-sectional and longitudinal studies. Secondly, we optimized the personal exposure levels by integrating activity patterns and infiltration factors to accurately evaluate the individual exposure levels. Thirdly, we explored the potential bio-mechanism for ozone related renal function declination through the causal mediation analysis. Our results will also provide reference for further related mechanism research.

However, there are still several limitations currently which need to be further improved. Firstly, the long-term association between ozone exposure and renal function was based on a cross-sectional study within only one city, which served a weak ability to examine the causal association liken to cohort study. Our current participants were selected from the WCDCS by stratified cluster random sampling, but some characteristics differed between the included and excluded groups (Table S5), which may lead to selection bias. Then, the ozone exposure concentrations of WCDCS were directly obtained from the CHAP dataset, which will overestimate the actual exposure levels. We calculated personal exposure concentrations in a later panel study. Our results showed that ambient concentration was about three times larger than individual value. Hence, we should develop more accurate exposure model for individuals in future research. Thirdly, ozone concentrations are strongly influenced by seasonal factors and are typically higher in summer. However, the current panel study was mainly conducted in autumn and winter, when ambient ozone pollution was light. Hence, we failed to reveal the effects of ozone exposure in a larger concentration range.

## 5 Conclusion

Our current research supplied evidence for the adverse effect of ozone pollution on renal function with chronic or acute exposure. The drinkers showed much more vulnerable to ozone exposure. Additionally, our findings suggested that hyperglycemia and insulin resistance might be the plausible bio-pathways for ozone exposure related renal function declination. Further research was needed to elucidate the potential mechanisms.

## CRediT authorship contribution statement

**Shouxin Peng**: Methodology, Software, Formal analysis, Writing − original draft. **Bingbing Chen**: Formal analysis, Writing − original draft. **Zhaoyuan Li**: Methodology, Visualization. **Jinhui Sun**: Data curation, Resources. **Feifei Liu**: Conceptualization, Software. **Xiaoyi Yin**: Visualization, Resources. **Yi Zhou**: Data curation, Software. **Huanfeng Shen**: Methodology, Funding acquisition, Writing − review & editing. **Hao Xiang**: Methodology, Project administration, Writing − review & editing, Funding acquisition.

## Declaration of Competing Interest

The authors declare that they have no known competing financial interests or personal relationships that could have appeared to influence the work reported in this paper.

## Data Availability

The authors do not have permission to share data.
