# Supplementary material for: Ambient ozone pollution impairs glucose homeostasis and contributes to renal function decline: Population-based evidence
Source: Ecotoxicol Environ Saf. 2024 Jan 1;269:115803. doi: 10.1016/j.ecoenv.2023.115803 (PMC10790241; doi:10.1016/j.ecoenv.2023.115803)
Supplement: Supplementary file 1 — Supplementary material [file mmc1.pdf]

Supplement Information

Contents

- Supplement eMethods
- Table S1. Previous evidence for the association between ozone pollution and kidney disease or renal function.
- Figure S1. Flow diagram of participants recruitment for WCDCS.
- Figure S2. Distribution for the research participants.
- Table S2. Baseline characteristics for the longitudinal panel study participants in Wuhan.
- Table S3. The lag effect for short-term ozone pollution on eGFR. Estimates and 95%CI per IQR (19.02 $\mu g/m^3$) increment. ($mL/min/1.73m^2$)
- Table S4. Association between long-term ozone exposure and renal function decline (eGFR < 60 $mL/min/1.73m^2$). ORs and 95%CI per 1 $\mu g/m^3$ increment.
- Table S5. The characteristics of the study participants excluded and the differences between included and excluded participants.

Supplement eMethods

1. Study design and participants

Current longitudinal panel study was conducted in Wuhan from September 2019 to January 2020. Specifically, participants were limited to healthy students aged 18-30 who live within 1 km of the School of Medicine, Wuhan University, have lived in the current residence for more than two years, and have no plans to leave during the study period. The exclusion criteria for students were: (i) smoked or with a history of alcohol abuse; (ii) clinical diagnosis of chronic diseases such as cardiopulmonary disease; (iii) diagnosed with infectious diseases or used anti-inflammatory drugs, antibiotics, or other drugs in the past one month. A group of 70 healthy adults were ultimately included. All the participant was recruited to complete a baseline questionnaire to collect demographic characters (i.e., age, gender, weight, height, etc.). And then, participants were divided into two groups to complete eight clinical visits from 9 Sep. 2019 to 7 Jan. 2020 and the interval was controlled at 1-2 weeks. At each follow-up visit, 10 mL of venous blood was collected from the median elbow vein of each study subject by a trained nurse before 8:00 a.m. All the 533 blood samples were centrifuged at 2500 rpm for 15 min, after which the separated plasma, serum and hemocyte were transformed into Eppendorf tubes (1.5 mL) and stored at -80 ◦C.

2. Statical analysis

2.1 Linear mixed-effect regression model

Short-term association between ozone pollution and eGFR was evaluated the linear mixed-effect (LME) model. LME model allows each subject to act as his or her own control over time and adjusts for between-subject covariates that do not vary over time. And the fixed and random effects of the LME model are estimated with the restricted maximum-likelihood method. The equation is as follows.

$$Y_{ij} = \beta_0 + \mu_i + \beta_{O_3} O_{3_{ij}} + \beta_1 X_{1_{ij}} + \cdots + \beta_p X_{p_{ij}} + \varepsilon_{ij}$$

where *i* and *j* denoted the participant and visit time; Y_{ij} was the blood lipid level; β_0 was the intercept for the population mean; μ_i represented the subject-specific random intercept; $\beta_1 X_{1ij}$ to $\beta_p X_{pij}$ indicated the potential confounding variables; $\beta_{O3} O_3$ was personal O_3 concentrations over time windows; ε_{ij} was the within-subject error term.

2.1 Mediation analysis

We performed a causal mediation analysis following the Baron-Kenny's step to examine the potential bio-mechanisms. Two LME models were built for the mediation analysis, one fitting for the ozone-mediator association and the other one fitting for the mediator-eGFR association (Equations 1, 2).

$$M_{ij} = \beta_0 + u_i + \beta_{O_3} O_{3_{ij}} + \beta_1 X_{1_{ij}} + \cdots + \beta_p X_{p_{ij}} + \varepsilon_{ij} \quad [1]$$

$$Y_{ij} = \gamma_0 + g_i + \gamma_{O_3} O_{3_{ij}} + \gamma_M M_{ij} + \gamma_1 X_{1_{ij}} + \cdots + \gamma_p X_{p_{ij}} + \eta_{ij} \quad [2]$$

In both of two equations, β_0 and γ_0 correspond to the intercept for the population mean; *ui* and *gi* correspond to the subject-specific random intercept. M_{ij} correspond to the potential mediators and

Y_{ij} correspond to eGFR measured for an individual i (i = 1, …, 70) at visit j (j = 1, …, 8). X_{1ij} to X_{pij} represent the priori-selected covariates, and ε_{ij} and η_{ij} represent the within-subject error term. γ_{O3} represents the natural direct effect (NDE), and the natural indirect effect (NIE) could be given by $\beta_{O3} \times \gamma_M$. The proportion mediated, which means the percentage of NIE over the total effect, was calculated by (NIE/(NIE + NDE)). All the above operations were performed using the "mediation" package of R software with bootstrapping of 1,000 simulations.

3. Exposure measurement

Long-term ambient fine particulate matter ($PM_{2.5}$) concentration were also derived from the ChinaHighAirPollutants (CHAP) datasets with temporal and spatial resolutions of 1 day and 1 km, respectively [1]. Detailed operations were similar to the ozone exposure assessment. Residential greenness was estimated by the vegetation index Normalized Vegetation Index (NDVI), which was quantified by satellite images from the 16-day Moderate Resolution Imaging Spectroradiometer product (https://lpdaac.usgs.gov/products/mod13q1v006/). We selected the greenness exposure within a 250 m buffer for analyses, as the influence of greenness coverage on the surrounding environment is typically limited to a range of 500 m [2]. Short-term individual $PM_{2.5}$ concentrations, temperature (T), and relative humidity (RH) were collected from the personal $PM_{2.5}$ monitor (Ai100, Huawei Technologies Co. Ltd., China), which is portable and applicable for monitoring real-time (hourly) individual exposure [3].

References

1. Wei J, Li ZQ, Lyapustin A, Sun L, Peng YR, Xue WH, Su TN, Cribb M. Reconstructing 1-km-resolution high-quality $PM_{2.5}$ data records from 2000 to 2018 in China: spatiotemporal variations and policy implications. ***Remote Sens Environ***. 2021; 252: 112136.
2. Pan M, Liu F, Zhang K, Chen Z, Tong J, Wang X, Zhou F, Xiang H. Independent and interactive associations between greenness and ambient pollutants on novel glycolipid metabolism biomarkers: A national repeated measurement study. ***Environ Res***. 2023; 233: 116393.
3. Peng SX, Sun JH, Liu FF, Li ZY, Wu CX, Xiang H. The effect of short-term fine particulate matter exposure on glucose homeostasis: A panel study in healthy adults. ***Atmos Environ***. 2021; 268: 118769.

Table S1. Previous evidence for the association between ozone pollution and kidney disease or renal function.

Authors	Study design	Ozone pollution	Kidney events	Main fundings
(Li et al., 2022) [1] Cross-sectional study	80,225 participants aged 30-79 years from the baseline data of the China Multi-Ethnic Cohort study.	79.2μg/m^3	CKD	An increase of 10 μg/m^3 in O_3 (OR = 1.10, 95%CI: 0.81 to 1.50) concentration was not associated with CKD.
(Paoin et al., 2022) [2] Cohort study	1,839 participants aged 52-71 years from the EGAT study in Thailand.	25.4 ppb	eGFR	An increase of 12 ppb in O_3 (β = -0.35%, -2.18% to 1.51%) concentration was not associated with eGFR.
(Yang et al., 2022) [3] Cross-sectional study	47,086 participants aged 49.2 (15.2) years from CNSCKD survey in China.	91.8μg/m^3	CKD	A 10 μg/m^3 increment in O_3 concentration was associated with an increased risk of CKD (1.11, 1.03 to 1.21).
(Lee et al., 2022) [4] Time-series study	Daily counts of emergency room admission for kidney disease from Jan. 2003 to Dec. 2013 in South Korea.	70.5μg/m^3	Total kidney disease, AKI	Emergency room visits for total kidney disease (1.01, 1.00 to 1.01) and AKI was associated with O_3.
(Hwang et al., 2021) [5] Cohort study	164,093 adults aged at least 40 years from the Korean National Health Insurance Service National Sample Cohort.	0.018 ppm	CKD	No significant difference (HR = 1.15, 95%CI: 0.86 to 1.54) compared to the 1st quantile group.
(Liu et al., 2022) [6] Cohort study	90,032 older adults from the Tianjin Chronic Disease Cohort in China.	66.10 μg/m^3 and 63.75 μg/m^3	CKD	Negative association between O_3 and CKD, with the HR of 0.275 (0.264 to 0.286) at 4th quantile concentrations compare to the 1st quantile.
(Mehta et al., 2023) [7] Cohort study	61,300,754 beneficiaries aged at least 65 years from Medicare Part A fee-for-service in US.	45.2ppb	AKI	O_3 pollution was positively associated with AKI, with HR of 1.03 (1.02 to 1.04) for a 10-ppb increase in warm-season O_3.
(Wen et al., 2023) [8] Cohort study	8,996 participants aged 50.97 years from CHCN-BTH cohort in China.	122.63 μg/m^3	eGFR	O_3 pollution was beneficial for eGFR with 1.151 mL/min/1.73m^2 (0.813 to 1.489) increment for 5.03 μg/m^3 increase in O_3 concentrations.
(Guo et al., 2022) [9] Cohort study	10,942 children and adolescents aged 18.1 years from two cohort in Chinese Taiwan and Chinese Hong Kong.	51.1μg/m^3	CKD	O_3 pollution was negatively associated with CKD, with HR of 0.81 (0.67 to 0.98) for a 10 μg/m^3 increase O_3 concentrations.
(Weaver et al., 2019) [10] Cross-sectional study	5,090 African Americans aged 55.4 years from Jackson Heart Cohort in US.	40.7 ppb	eGFR	3-year O_3 concentrations were inversely associated with eGFR (β = -0.3, 95%CI: -0.6 to -0.04).
(Chang et al., 2022) [11] Cohort study	5,301 CKD patients aged 62.60 years were recruited from Chinese Taipei.	27.16 ppb	Renal progression (eGFR decreased more than 25% from the baseline)	No significant association between O_3 and renal progression, with the HR of 0.90 (0.75 to 1.09) at 4th quantile concentrations compare to the 1st quantile.

Abbreviation: CKD, chronic kidney disease; O_3, ozone; OR, odds ratio; CI, confidence interval; eGFR, estimated glomerular filtration; AKI, acute kidney injury; HR, hazard ratio.

Reference:

1. Li S, Meng Q, Laba C, Guan H, Wang Z, Pan Y, Wei J, Xu H, Zeng C, Wang X, Jiang M, Lu R, Guo B, Zhao X. Associations between long-term exposure to ambient air pollution and renal function in Southwest China: The China Multi-Ethnic Cohort (CMEC) study. **Ecotoxicol Environ Saf 2022**, 242**:** 113851.
2. Paoin K, Ueda K, Vathesatogkit P, Ingviya T, Buya S, Dejchanchaiwong R, Phosri A, Seposo XT, Kitiyakara C, Thongmung N, Honda A, Takano H, Sritara P, Tekasakul P. Long-term air pollution exposure and decreased kidney function: A longitudinal cohort study in Bangkok Metropolitan Region, Thailand from 2002 to 2012. **Chemosphere 2022**, 287(Pt 1)**:** 132117.
3. Yang C, Wang W, Wang Y, Liang Z, Zhang F, Chen R, Liang C, Wang F, Li P, Ma L, Li S, Deng F, Zhang L. Ambient ozone pollution and prevalence of chronic kidney disease: A nationwide study based on the China National survey of chronic kidney disease. **Chemosphere 2022**, 306**:** 135603.
4. Lee W, Prifti K, Kim H, Kim E, Yang J, Min J, Park JY, Kim YC, Lee JP, Bell ML. Short-term Exposure to Air Pollution and Attributable Risk of Kidney Diseases: A Nationwide Time-series Study. **Epidemiology 2022**, 33(1)**:** 17-24.
5. Hwang SY, Jeong S, Choi S, Kim DH, Kim SR, Lee G, Son JS, Park SM. Association of Air Pollutants with Incident Chronic Kidney Disease in a Nationally Representative Cohort of Korean Adults. **Int J Environ Res Public Health 2021**, 18(7).
6. Liu H, Shao X, Jiang X, Liu X, Bai P, Lin Y, Chen J, Hou F, Cui Z, Zhang Y, Lu C, Liu H, Zhou S, Yu P. Joint exposure to outdoor ambient air pollutants and incident chronic kidney disease: A prospective cohort study with 90,032 older adults. **Front Public Health 2022**, 10**:** 992353.
7. Mehta AJ, Zanobetti A, Bind MA, Kloog I, Koutrakis P, Sparrow D, Vokonas PS, Schwartz JD. Long-Term Exposure to Ambient Fine Particulate Matter and Renal Function in Older Men: The Veterans Administration Normative Aging Study. **Environ Health Perspect 2016**, 124(9)**:** 1353-1360.
8. Wen F, Xie Y, Li B, Li P, Qi H, Zhang F, Sun Y, Zhang L. Combined effects of ambient air pollution and PM(2.5) components on renal function and the potential mediation effects of metabolic risk factors in China. **Ecotoxicol Environ Saf 2023**, 259**:** 115039.
9. Guo C, Chang LY, Wei X, Lin C, Zeng Y, Yu Z, Tam T, Lau AKH, Huang B, Lao XQ. Multi-pollutant air pollution and renal health in Asian children and adolescents: An 18-year longitudinal study. **Environ Res 2022**, 214(Pt 4)**:** 114144.
10. Weaver AM, Wang Y, Wellenius GA, Young B, Boyle LD, Hickson DA, Diamantidis CJ. Long-term exposure to ambient air pollution and renal function in African Americans: the Jackson Heart Study. **J Expo Sci Environ Epidemiol 2019**, 29(4)**:** 548-556.
11. Chang PY, Li YL, Chuang TW, Chen SY, Lin LY, Lin YF, Chiou HY. Exposure to ambient air pollutants with kidney function decline in chronic kidney disease patients. **Environ Res 2022**, 215(Pt 2)**:** 114289.

Figure S1

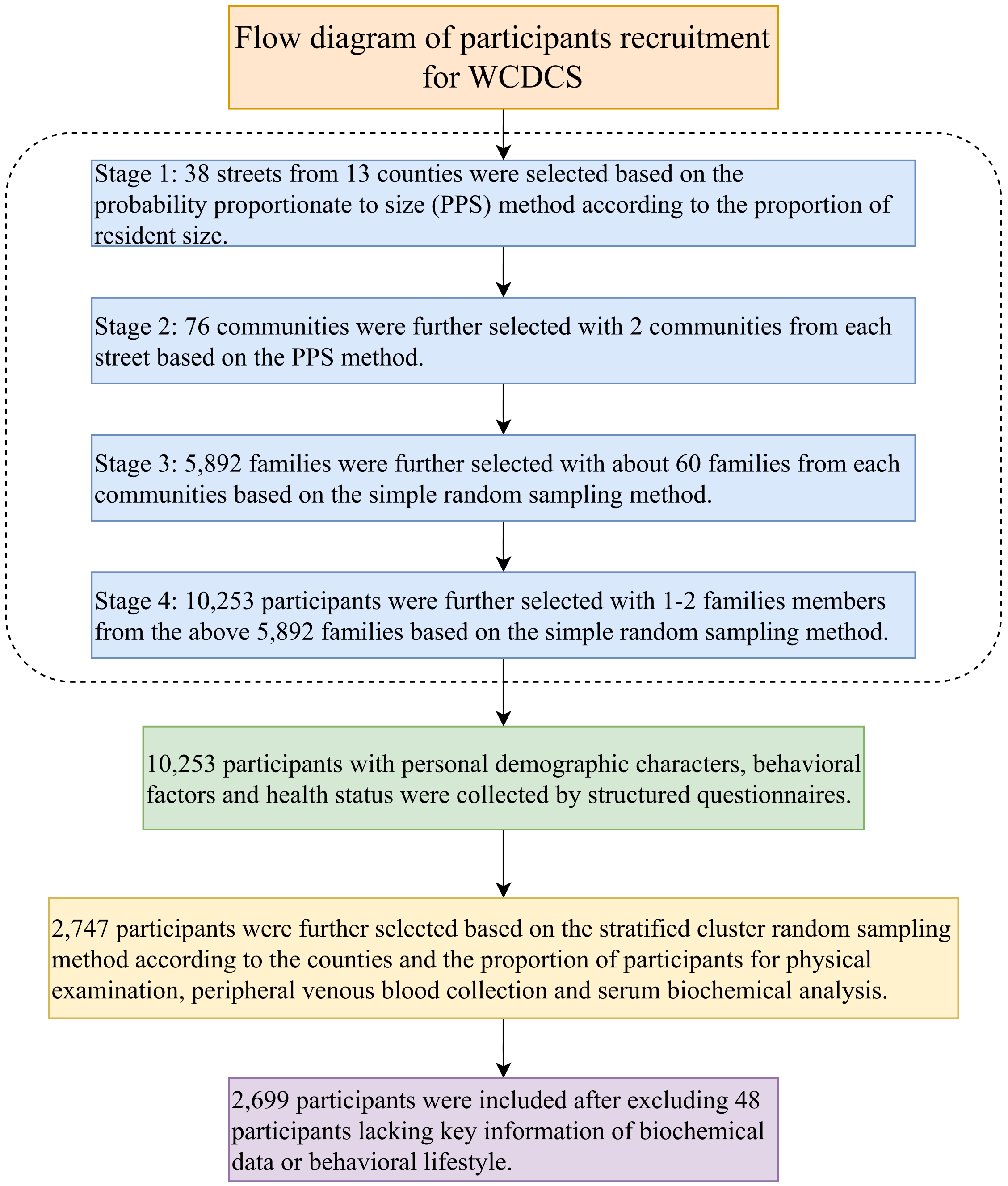
Flow diagram of participants recruitment for WCDCS
Stage 1: 38 streets from 13 counties were selected based on the probability proportionate to size (PPS) method according to the proportion of resident size.
Stage 2: 76 communities were further selected with 2 communities from each street based on the PPS method.
Stage 3: 5,892 families were further selected with about 60 families from each communities based on the simple random sampling method.
Stage 4: 10,253 participants were further selected with 1-2 families members from the above 5,892 families based on the simple random sampling method.
10,253 participants with personal demographic characters, behavioral factors and health status were collected by structured questionnaires.
2,747 participants were further selected based on the stratified cluster random sampling method according to the counties and the proportion of participants for physical examination, peripheral venous blood collection and serum biochemical analysis.
2,699 participants were included after excluding 48 participants lacking key information of biochemical data or behavioral lifestyle.

Figure S2

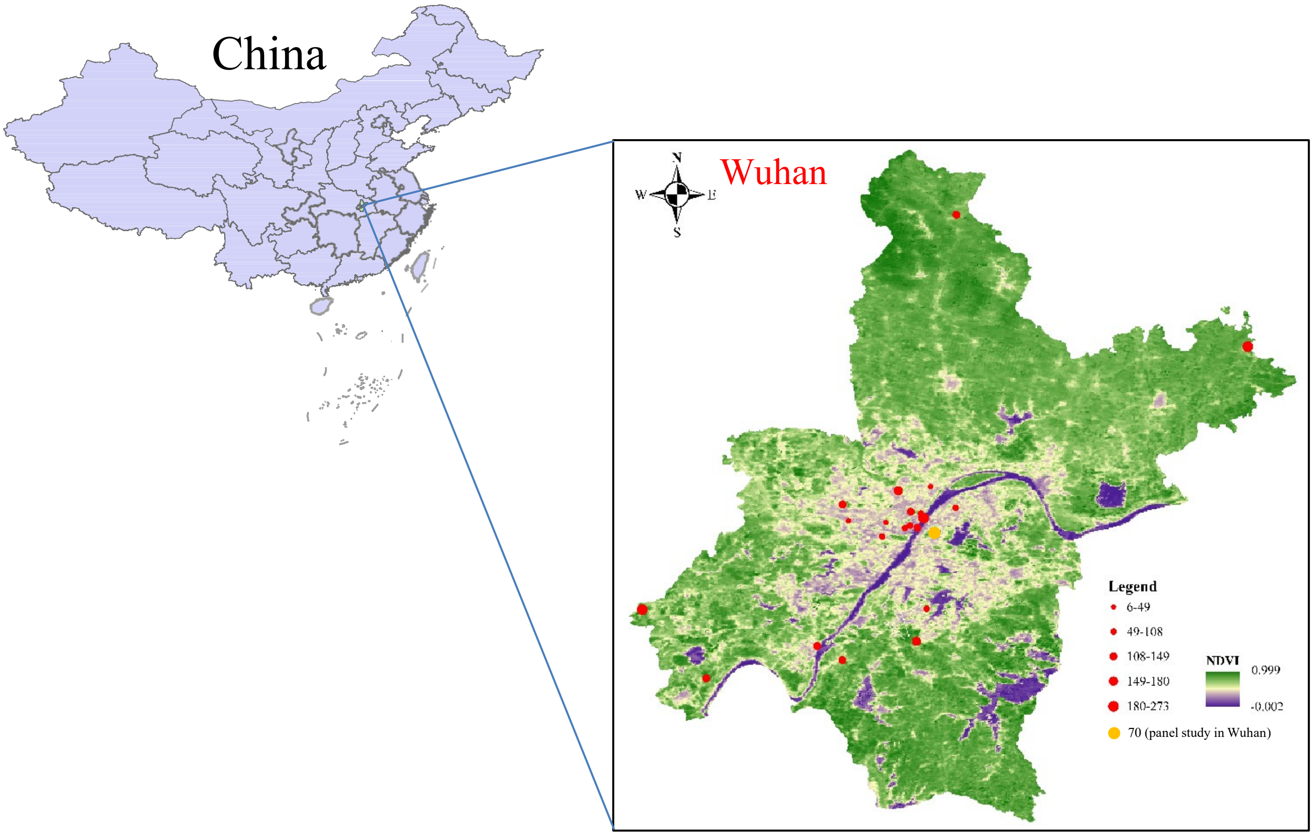
China
Wuhan
N
W
E
S
Legend
6-49
49-108
108-149
149-180
180-273
70 (panel study in Wuhan)
NDVI
0.999
-0.002

Table S2. Baseline characteristics for the longitudinal panel study participants in Wuhan.

Characters	Mean (SD) or n (%)
Age, years	20.4 ± 1.53
Gender	
Male	14 (20%)
Female	56 (80%)
BMI, kg/m^2	21.50 ± 2.75
eGFR	117.90 ± 15.70
Fasting plasma glucose	4.40 ± 0.456
Triglyceride	1.00 ± 0.45
TyG index	0.71 ± 0.38
Systolic pressure	111.03 ± 9.29
Diastolic pressure	76.99 ± 6.55
Sleep duration, hours	7.82 ± 1.16
Ambient ozone, μg/m^3	48.20 ± 40.00
Personal ozone, μg/m^3	16.00 ± 14.70
Outdoor time, hours	3.08 ± 1.81

Abbreviations: SD, standard deviation; BMI, body mass index; eGFR, estimated glomerular filtration rate; TyG index, triglyceride-glucose index.

Table S3. The lag effect for short-term ozone pollution on eGFR. Estimates and 95%CI per IQR (19.02 μg/m^3) increment. (mL/min/1.73m^2)

	lag 1 day	lag 2 day	lag 3 day
Model 1[a]	-2.36 (-3.52, -1.20)	-2.87 (-4.16, -1.59)	-2.58 (-3.91, -1.27)
Model 5[b]	-2.09 (-3.28, -0.90)	-2.76 (-3.92, -1.60)	-2.51 (-3.70, -1.33)
+$PM_{2.5}$	-2.19 (-3.46, -0.93)	-2.24 (-3.67, -0.82)	-2.41 (-3.74, -1.08)
+AT	-3.29 (-4.90, -1.69)	-3.34 (-5.08, -1.60)	-3.40 (-4.97, -1.82)
+RH	-2.28 (-3.48, -1.09)	-2.81 (-4.15, -1.48)	-2.70 (-3.90, -1.51)

Abbreviation: eGFR, estimated glomerular filtration rate; IQR, interquartile range; CI, confidence interval; $PM_{2.5}$, fine particulate matter (μg/m^3); AT, ambient temperature (°C); RH, relative humidity (%).

[a] Model 1 was the crude model.

[b] Model 5 adjusted for age, gender, BMI, physical exercise, smoking, drinking and sleep duration.

Table S4. Association between long-term ozone exposure and renal function decline (eGFR < 60 mL/min/1.73m^2). ORs and 95%CI per 1 μg/m^3 increment.

	ORs (95%CI)
Ave 1-year	1.08 (1.03, 1.13)
Ave 2-year	1.06 (1.03, 1.10)
Ave 3-year	1.06 (1.02, 1.10)

Abbreviations: eGFR, estimated glomerular filtration rate; OR, odd ratio; CI, confidence interval. The logistic regression models were fully adjusted with age, gender, BMI, hypertension, diabetes, education status, smoking status, drinking status, occupation, and marriage status.

Table S5. The characteristics of the study participants excluded and the differences between included and excluded participants.

	Included participants	Excluded participants	χ^2	*P*-value*
No.	2,699	7,554		
Gender			152.91	<0.001
Male	970 (35.9%)	3,761 (49.8%)		
Female	1,729 (64.1%)	3,793 (50.2%)		
Age, years			465.8	<0.001
<45	676 (25%)	3,616 (47.9%)		
45-65	1,429 (52.9%)	3,056 (40.5%)		
≥65	594 (22%)	882 (11.7%)		
BMI, kg/m^2			20.63	<0.001
<24	1,225 (45.4%)	1,990 (26.3%)		
>24	1,474 (54.6%)	1,904 (25.2%)		
Missing	0 (0%)	3,660 (48.5%)		
Smoking			14.55	<0.001
Yes	534 (19.8%)	1,766 (23.4%)		
No	2,165 (80.2%)	5,788 (76.6%)		
Drinking			0.76	0.384
Yes	607 (22.5%)	1,763 (23.3%)		
No	2,092 (77.5%)	5,791 (76.7%)		
Education			166.77	<0.001
Middle or below	2,123 (78.7%)	4,926 (65.2%)		
College or above	576 (21.3%)	2,628 (34.8%)		

Notes: * The chi-square test excluded missing values.
